# Estimating the Impact of Covid-19 and Policy Responses on Australian Income Distribution Using Incomplete Data

**DOI:** 10.1007/s11205-021-02826-0

**Published:** 2021-10-26

**Authors:** Jinjing Li, Yogi Vidyattama, Hai Anh La, Riyana Miranti, Denisa M. Sologon

**Affiliations:** 1 NATSEM, University of Canberra, Canberra, Australia; 2 Luxembourg Institute of Socio-Economic Research, Luxembourg, Luxembourg

**Keywords:** Covid-19, Nowcasting, Income inequality, Australia, D31, H23

## Abstract

This paper undertakes a near real-time analysis of the income distribution effects of the Covid-19 crisis in Australia to understand the ongoing changes in the income distribution as well as the impact of policy responses. By semi-parametrically combining incomplete observed data from three different sources–the monthly Longitudinal Labour Force Survey, the Survey of Income and Housing and administrative payroll data–we estimate the impact of Covid-19 on the Australian income distribution and decompose its impact into the income shock effect and the policy effect between February and June 2020, covering the immediate periods before and after the initial Covid-19 outbreak. Our results suggest that, despite growth in unemployment, the Gini coefficient of equivalised household disposable income dropped by more than 0.02 points between February and June 2020. This reduction is due to the additional wage subsidies and welfare supports offered as part of the policy response, offsetting the increase in income inequality from the income shock effect. The results shows the effectiveness of temporary policy measures both in maintaining living standards and avoiding increases in income inequality. However, the heavy reliance on the support measures shown in the modelling raises the possibility that the changes in the income distribution may be reversed, or even that inequality and living standards could substantially worsen once the measures are withdrawn.

## Background

The Covid-19 pandemic and the associated government response led to a significant shift in the usual pattern of social and economic activities. Many governments worldwide imposed various measures in the hope of containing the outbreak. Both the pandemic and the policy measures have had wide-reaching and highly asymmetric implications for many aspects of life in most countries (Baker et al., 2020; Bonaccorsi et al., 2020; McKibbin & Fernando, 2021). In Australia, all international travel has been banned since 20 March 2020, and the government has imposed extended lockdowns of Australian businesses during periods of rapid virus spread. 1The border closure and lockdowns have, without a doubt, had a significant impact on the economy, reflected in elevated unemployment levels. According to the National Skill Commission (2020), 17 of 19 industries recorded reductions in employee jobs between mid-March and the end of May 2020.

Besides the loss of income and job opportunities, the Covid-19 shock also has implications for the distribution of income. As well as the economic importance of studying income inequality, economic inequality may also affect the outcome of Covid-19 policies due to economic segregation and decreased social mobility (Oronce et al., 2020). While members of the community could feel the changes in economic activity almost immediately after the initial spread of Covid-19, the statistics needed to understand the current state of the economy are often limited and are only available with a significant time lag (Chetty et al., 2020). Detailed household information is rarely available promptly given that most household surveys are collected annually (or less frequently) due to the substantial cost involved. Thus, there may be a non-negligible difference between the current population and the one reflected in the collected data. Such a discrepancy limits the capability to analyse sudden events, including the spread of Covid-19.

To overcome this gap, an increasing number of studies have resorted to adapting the *nowcasting* technique to represent statistics for the present state, the recent past and the near future by updating the latest collected data with up-to-date external statistics or controls (Bańbura et al., 2013). While the idea originated in meteorology, economists have adapted the technique to estimate timely economic indicators (Giannone et al., 2008). More recently, nowcasting techniques have been applied to produce current estimates of poverty and income inequality. Some research has used inflation indexation and proportional adjustment of industry-specific employment rates combined with a tax-benefit simulator to evaluate the policy impact of various tax-benefit rules (Navicke et al., 2014). Previous studies have demonstrated the applicability and transferability of this technique across countries (Kuzmenko and Roienko, 2017) and the technique’s usefulness in incorporating newer data. This feature has become more relevant in the context of the Covid-19 pandemic, as it enables researchers to construct a near real-time household sample for income analyses.

This paper contributes to the literature in three ways. First, the paper empirically tests the applicability of the nowcasting technique in Australia. We show that our proposed method can construct a near real-time household sample for income analyses from partially observed data, capturing the short-term economic changes and resembling the official statistics. Second, we provide the first estimates of the effects of the Covid-19 on the income distribution in Australia and show evidence of the heterogeneous effects of the pandemic based on individual-level data. Third, we estimate the contribution of the government response in stabilising the income distribution, showing the importance of this response and the level of reliance on the temporary measures. Given the relative success of Covid-19 containment in Australia compared with most other Western countries, the examination of the changes in Australian income inequality could also contribute to the understanding of broader pandemic management policies worldwide, especially in light of the association between inequality and disease spread.

## Nowcasting and its Benefits in Times of Crisis

Modelling the socio-economic consequences of a shock is vital to inform policy decision-making (Bok et al., 2018). The Covid-19 pandemic and the subsequent restrictions to control its spread have not only caused a downturn in economic performance marked by negative GDP growth in many countries (McKibbin and Fernando, 2021), but is likely to affect levels of poverty and income inequality (Bonaccorsi et al., 2020). Given how widespread Covid-19 is globally and how restrictive social measures are, it is crucial to understand the impact of the crisis on inequality and poverty in a timely manner in order to formulate appropriate policy responses.

Historically, major economic shocks tend to induce changes in income inequality. In the 1997–1999 Asian financial crisis, South Korea was one of the worst-hit economies among developed countries, with contracted GDP, rising unemployment and sharply worsened income inequality (Cheong, 2001). The 2008 global financial crisis severely affected all EU Member States, with a plunge in real GDP ranging from 5 per cent in Cyprus to 40 per cent in Latvia (De Beer, 2012). Unemployment rose rapidly, and the governments in the EU introduced fiscal interventions with unemployment and welfare benefits. Nevertheless, income inequality increased in many countries. Although most EU countries started to recover in the new decade, many governments had accumulated substantial debt (Matsaganis and Leventi, 2014). This highlights the need for the governments to come up with a well-targeted policy as well as a careful allocation of resources to avoid plunging into austerity after an initial stimulus (Matsaganis and Leventi, 2014).

A few European countries have analysed the impact of Covid-19 based on the nowcast approach. Examples include Beirne et al. (2020) and O’Donoghue et al. (2020) for Ireland, Figari and Fiorio (2020) for Italy, Sologon et al. (2020) for Luxembourg, and Brewer and Tasseva (2021) and Bronka et al. (2021) for the UK. In the case of the UK, earnings subsidies were found to cushion household income across the entire distribution, providing the primary insurance mechanism against the adverse income shock; existing tax-benefit rules were found to complement this scheme in providing social protection. In the case of Ireland, O’Donoghue et al. (2020) found that the crisis-induced income-support policy responses (wage subsidies, pandemic-related unemployment and sickness benefits) combined with existing progressive elements of the tax-benefit system were effective in counteracting the increase in income inequality in the early months of the pandemic, leading to a drop in income inequality. In Italy, Figari and Fiorio (2020) estimated that income inequality grew despite the policy response and the existing stabilisers built into the tax-benefit system.

The existing nowcast approaches used, however, rely heavily on parametric assumptions regarding changes in the labour market. The analyses tend to assume the changes both in the extensive and intensive margin are either stochastic (e.g., Brewer and Tasseva, 2021; Figari and Fiorio, 2020) or based on the parametric regressions derived from the pre-crisis household survey (e.g., O’Donoghue et al., 2020). The implicit assumption that the labour market shocks are equally random for everyone in the same industry may not always hold, especially when shocks are expected to have highly heterogeneous impacts across the population.

To address this limitation, this paper proposes a semi-parametric nowcast approach combining multiple incomplete microdata as well as administrative statistics for estimations of the income distribution; this approach exploits the availability of both a comprehensive household survey as well as a shorter monthly labour force survey in Australia. Our focus in this paper is the short-term (3 months) impact in the early phase of the pandemic and the associated policy response. We propose a calibrated income distribution estimation based on: (i) actual data on changes in employment (both at the extensive and intensive margin) drawn from the up-to-date monthly Longitudinal Labour Force Survey (LLFS); (ii) administrative data on the changes in earnings at the industry and age group level drawn from the Weekly Payroll Jobs and Wages in Australia dataset; and (iii) the Survey of Income and Housing (SIH), which is a comprehensive biannual household income survey collected in 2017–18.

## Data

As a comprehensive household survey is not available to capture the pre- and post-pandemic changes in the income distribution, we reconstruct the income distribution profiles by combining these three different datasets–the LLFS, the weekly payroll statistics and the SIH–that describe the evolution of the labour market in Australia. All datasets are collected by the Australian Bureau of Statistics (ABS).

The LLFS provides details on geography, demography (e.g., age, sex, educational attainment, family characteristics), labour market participation (e.g., hours worked, industry, occupation, details of the last job) and transitions into and out of the employment of Australians (Australian Bureau of Statistics, 2020b). The data, however, does not contain any income information. We use the waves between February 2020 and June 2020 (5 waves), covering the periods immediately before and after Covid-19 began to spread in Australia. The selection of the data period allows us to capture the effect of Covid-19 progression in Australia.

The second data source we use, the SIH 2017–18, collects data on demographics (e.g., sex, age, marital status), housing, labour force characteristics, education, income, assets and childcare (Australian Bureau of Statistics, 2019). At the individual level, the SIH provides detailed information for those living in private dwellings on labour force status, the number of jobs currently held, weekly hours worked and duration of unemployment as well as detailed income information. Given the overlapping variables between the LLFS and the SIH, we are able to match the observed characteristics in the two surveys to examine the likely impact of Covid-19.

Table 1 reports the mean values of key socio-economic variables in the LLFS and the SIH. In terms of demographics, the LLFS and SIH populations are very similar, although the LLFS population appears to be slightly older as reflected by the proportion of the population aged 85 or above. With regard to employment, the proportion of the population unemployed is slightly lower in the February 2020 LLFS than in the 2017–18 dataset. However, we can see a clear trend of deteriorating employment numbers since the start of the pandemic.

**Table 1:** Mean values of selected demographic and employment variables

Variable	SIH 17–18	LLFS Feb 20	LLFS Mar 20	LLFS Apr 20	LLFS May 20	LLFS Jun 20
Age and Marriage						
Age (under 85)	44.7	44.7	44.7	44.7	44.7	44.8
Age 85 or above	1.8%	2.0%	1.9%	2.0%	2.0%	2.0%
Married	61.0%	59.4%	59.6%	59.9%	59.9%	60.0%
Education						
Postgraduate	9.4%	10.2%	10.1%	10.2%	10.5%	10.7%
Bachelor	18.4%	18.4%	18.6%	18.6%	18.2%	18.4%
Certificate	30.3%	27.1%	27.0%	26.6%	26.2%	26.1%
Year 12	16.0%	17.1%	17.0%	17.2%	17.3%	17.2%
Others	25.8%	27.2%	27.3%	27.3%	27.7%	27.6%
Employment Status						
Full-time	41.0%	43.8%	43.2%	41.9%	41.5%	41.3%
Part-time	21.9%	19.9%	20.2%	18.5%	17.8%	18.9%
Unemployed	3.6%	3.7%	3.7%	4.1%	4.4%	4.7%
Not in labour force	33.4%	32.5%	32.9%	35.5%	36.3%	35.2%
Employment Status						
Not employed	37.1%	36.2%	36.6%	39.6%	40.7%	39.8%
Employee	52.9%	53.2%	53.2%	50.6%	49.3%	50.3%
Self-employed and others	10.1%	10.5%	10.2%	9.8%	10.0%	9.8%

To refine the modelling of the heterogeneous shock which affects earnings across different industries, occupations and demographic profiles, we incorporate the longitudinal Weekly Payroll Jobs and Wages dataset. This is sourced from administrative data, covering businesses with Single Touch Payroll (STP)-enabled payroll or accounting software, and which is provided to the Australian Taxation Office (ATO). Since July 2019, all employers in Australia have been covered by STP unless an exemption has been granted. This data includes changes in payroll jobs, changes in total wages paid and changes in average weekly wages per job (Australian Bureau of Statistics, 2020a).

The three datasets can be understood as different projections of the socio-economic profile of Australia, although each alone does not provide sufficient information for our analysis. The combination of the three sources of data can offer a more complete picture of the socio-economic changes we are examining. Additionally, as information related to changes in the labour market – the primary location for the shocks from Covid-19 – is already captured in the data, there is less need to use specific parametric equations to model the evolution of the labour market, thus avoiding many restrictive assumptions. The use of multiple unit record datasets enables us to reconstruct the socio-economic profile semi-parametrically to reflect the observed changes due to Covid-19, in contrast with most of the existing nowcast approaches in the literature.

## Methodology

### General Estimation Strategy

We focus on the short-term change in income distribution given the data availability and the typical use case of nowcasting. We also assume that factors other than Covid-19, such as demographic change and other unpredicted events, are negligible in reshaping the income distribution in the immediate periods after the onset of the pandemic. The changes in the income distribution can therefore be decomposed into two main parts: the change induced by the income shock (part A) and the government policy response (part B):

1 $$\begin{array}{*{20}{l}}{\Delta I = I\left( {{p_1},{y_1}} \right) - I\left( {{p_0},{y_0}} \right)}\\{\;\;\;\;\; = \underbrace {I\left( {{p_0},y_1^*~} \right) - I\left( {{p_0},{y_0}} \right)}_{Income~Shock~Effect~\left( A \right)} + \underbrace {I\left( {{p_1},{y_1}} \right) - I\left( {{p_0},y_1^*} \right)}_{Policy~{\mathop{\rm Re}\nolimits} sponse~Effect~\left( B \right)}}\end{array}$$

where *I* is an outcome measure calculated from the entire income distribution (e.g., average disposable income, Gini coefficient), *p* is the government Covid-19 response policies and *y* is the distribution of gross market income. The subscript 0 refers to the pre-Covid baseline period, which is February 2020, and the subscript 1 refers to the periods after the initial spread of Covid-19, ranging between April and June 2020.^2^ We use ${y}_{1}^{*}$ to denote the unobserved gross market income distribution sans the policy intervention after the initial spread of Covid-19.

Equation (1) resembles a similar decomposition framework, which quantifies the contribution to inequality changes of the specific components between two points in time using a counterfactual simulation approach (Bargain & Callan, 2010; Bargain et al., 2017). Part A in Eq. (1) captures the changes in income distribution due to the changes in the market income. As Covid-19 dominated the income shock between Feb 2020 and June 2020, we assume that most (if not all) changes in Part A can be attributed to Covid-19. Part B captures the policy changes and their effects on income distribution. As all changes in the policies are part of the government response to Covid-19, the effect of B is also a measure of the effectiveness of the policy response in stabilising the market income shock via welfare policies. It should be noted that Eq. (1) does not incorporate the interaction term of the two major components, as the policy response can only be meaningfully interpreted conditional on the existence of Covid-19. Therefore, a sequential decomposition framework becomes more suitable in this particular analysis. The outcome measures in this paper include the mean and the Gini coefficient^3^ of gross market income as well as of equivalised disposable income.

The estimation of Eq. (1) replies on the estimation of three income distributions^4^: the pre-Covid income distribution $I\left({p}_{0},{y}_{0}\right)$, the Covid-19 income distribution $I\left({p}_{1},{y}_{1}\right)$, and the counterfactual Covid-19 income distribution without any policy response $I\left({p}_{0},{y}_{1}^{*}\right).$ We use the samples from February to estimate $I\left({p}_{0},{y}_{0}\right)$ and the samples from a month later than March to estimate $I\left({p}_{1},{y}_{1}\right)$. The more challenging part of the decomposition is the estimation of $I\left({p}_{0},{y}_{1}^{*}\right)$, where we need to simulate the likely outcome should there be no welfare policy intervention (${p}_{0}$) amid the Covid-19 market income shock (${y}_{1}$). The estimation may be difficult due to the nature of the shock as well as the complex behaviour responses associated with it. More specifically, we do not know what the extent of labour market demand would have been if the government had not provided subsidies that affected approximately 3.5 million jobs, accounting for one quarter of the labour force (Australian Treasury, 2020). To accommodate the possible behavioural and market responses, instead of providing a point estimate with a strong assumption, we estimate the upper and the lower bounds of $I\left({p}_{0},{y}_{1}^{*}\right)$ using two extreme estimates:

- Lower bound estimate: we assume no jobs will be lost should the government withdraw the Covid-19 related subsidies to employers.
- Upper bound estimate: we assume that all subsidised jobs would be lost should there be no subsidy.

Although neither estimate above is realistic, the range is sufficiently large to provide a robust estimate of the terms A and B in Eq. (1). The difference between the two estimates reflects the uncertainty in the behavioural response. It is likely that the most realistic counterfactual resides somewhere in between these two extreme estimates. The ABS (2020c) reported a reduction of nearly 600,000 in the number of people employed in the one-month period between March and April 2020. If this trend had continued, 3.5 million jobs would have been gone in six months’ time, presenting a troubling economic picture a few months into the pandemic without effective policy intervention. The halfway point between the lower and the upper bound estimates could also be considered as the approximation of a scenario in which limited success in containment had been achieved a few months into pandemic. Although it is numerically possible to estimate a median scenario with assumed job loss elasticity, the result from the simulation would be sensitive to assumptions about human behaviours for which we do not have sufficient evidence. These include, but are not limited to, changes in behaviour due to fear of virus contamination, changes in the decision-making processes for labour supply in a family, and the additional spatial constraints of business activities. We therefore focus on the boundaries of the estimates based on those assumptions that have major influences on the results rather than the variations introduced by the sampling process.

### Constructing Demographics, Labour Market and Income Distributions

To capture the changes in demographics and employment while retaining income information, we estimate a set of new weights for the latest SIH to mimic the demographic and employment patterns observed in the monthly LLFS, reflecting the changes in both intensive and extensive margins in the labour market. We follow the semi-parametric method developed by DiNardo et al. (1996) to adjust the weight of each observation in the 2017–18 SIH with an estimated ratio. Specifically, the weight of an individual $i$ from the SIH ($w_{{i,{\text{SIH}}}}$) is updated using the Bayesian rule:

2 $$\begin{array}{*{20}c} {w_{{i,{\text{SIH}} \to {\text{LLFS}}}} = w_{{i,{\text{SIH}}}} \frac{{{\text{Pr}}\left( {X_{i} {\text{|LLFS}}} \right)}}{{{\text{Pr}}\left( {X_{i} {\text{|SIH}}} \right)}} = w_{{i,{\text{SIH}}}} \frac{{{\text{Pr}}\left( {{\text{LLFS|}}X_{i} } \right)}}{{{\text{Pr}}\left( {{\text{SIH|}}X_{i} } \right)}}\gamma } \\ \end{array}$$

where ${\text{Pr}}\left({X}_{i}|{\text{LLFS}}\right)$ and ${\text{Pr}}\left({X}_{i}|{\text{SIH}}\right)$ are the conditional probabilities of observing the characteristics ${X}_{i}$ in the LLFS and SIH datasets, respectively; ${\text{Pr}}\left({\text{LLFS}}|{X}_{i}\right)$ and ${\text{Pr}}\left({\text{SIH}}|{X}_{i}\right)$ are the probabilities of observations with characteristics ${X}_{i}$ drawn from the LLFS and SIH, respectively. The characteristics vector $X$ contains:

- demographic characteristics (age, age squared, marital status, gender, overseas born);
- employment characteristics (19 industry groups and 8 occupation groups for working population, type of employment, usual hours of work, number of jobs, duration of unemployment, and the interactions with gender and age); and
- household characteristics (number of young children in different age groups, state or territory in which the household is located).

Appendix Table 7 contains further information about the model specification and the estimation results. Most (sets of) variables that are statistically significant are those related to employment, reflecting the differences in the employment profiles between datasets. The ratio $\gamma$ can be interpreted as the prior, which is the relative ratio of the dataset size. We adjust the ratio slightly to ensure the weighted total population in our model matches the expected national population, reflecting the population growth between the time of the SIH collection and 2020.

In addition to the distributional adjustment, we also index wage income by official Average Weekly Earnings (AWE) data, and we index investment income by an assumed annual growth rate of 2.5% on top of the Consumer Price Index (CPI) until February 2020. Childcare expenses, which are used to estimate post-childcare disposable income, are also indexed using the CPI. This allows the nominal income to match with the policy parameters we use in the tax-benefit simulation.

From March 2020 onwards, we capture the income volatility in wages using the payroll information by industry (2-digit ANZSIC code, 19 groups) and age group (7 groups). To reflect the volatile effect of Covid-19 on the financial market, we use the default portfolio performance of the largest pension fund in Australia (Australian Super, 2020) to index investment income between February and June 2020. Incomes from other sources are assumed to be stable over the three months we study.

### Modelling Policy Responses to the Covid-19 Crisis

In response to the Covid-induced economic shocks, the Australian government announced several temporary taxation and welfare benefit changes that directly affected employment and the income level of households, including a one-off payment for specific welfare recipients, increased unemployment and other benefits, temporary support for free childcare, and wage subsidies to employees via eligible employers.

In March 2020, the government announced a one-off economic support payment of A$1,500 to nearly all recipients of specific welfare payments, including pensions, unemployment benefits and Family Tax Benefit (FTB). The majority of recipients received the first payment ($750) by mid-April, and the second payment ($750) was made starting from mid-July. Besides one-off payments, the government also introduced a temporary payment of $550 per fortnight, known as the Coronavirus Supplement, to eligible welfare payment recipients. The unemployment benefit was also doubled to $1,115.70 per fortnight and its eligibility criteria were relaxed, with recipients not required to fulfil their usual job searching obligations. Additionally, the taper rate, which determines the reduction of the payment when a partner’s income exceeds a threshold, was also temporarily changed, and asset testing was suspended for selected benefits (Centrelink, 2020). Along with the changes in direct welfare payments, the government also provided full subsidies to childcare services for approximately three months from the start of April to the end of June. The measure was intended to help childcare providers stay open and keep employees in their jobs.

Additional to the increased welfare payments, the government also announced a wage subsidy package called “JobKeeper”, providing eligible employers with a flat payment of A$1,500 per fortnight per employee, irrespective of prior or current hours and earnings. To be eligible, employers had to show that their turnover had reduced by at least 50 per cent for large firms and 30 per cent for smaller firms during the pandemic (Australian Treasury, 2020). This payment also included self-employed people who could demonstrate a sizable financial downturn. The flat payment rate meant that many eligible part-time or long-term casual employees could earn more than their regular pay. A review from the Australian Treasury suggested that the JobKeeper program covered over 920,000 organisations and around 3.5 million individuals during April and May 2020, with total payments exceeding A$20 billion (Australian Treasury, 2020).^5^

To estimate the impact of the policy changes, we use the latest version of an Australian tax-transfer microsimulation model, STINMOD + , to calculate household disposable income based on the corresponding tax and social transfer rules, including the changes in direct welfare payments and the childcare subsidy.^6^ As the Australian welfare system is highly means-tested, and the vast majority of the eligibility conditions do not depend on previous contributions or complex employment history, we can estimate welfare payments with a relatively high degree of accuracy. As the take-up rate of means-tested benefits in Australia is generally high, with relatively low stigma (Mood, 2006), we assume full take-up in our simulation. The temporary stimulus payments (and the expected changes) have been annualised to better reflect the general trend of income and the fact that some of these additional payments may not have arrived in a timely way for many households due to the prolonged administrative process.

To reflect the relaxed job search requirement for the unemployment benefit, all individuals losing their jobs since February are assumed to be eligible for the unemployment benefit as per the policy change. We use the LLFS to estimate the propensity of being employed in any wave since February prior to being out of labour force with a standard probit model (see Appendix Table 8 for the model specification) and apply the coefficients to the SIH data to simulate the additional eligibility for unemployment benefit for those who are out of the labour force.

As we do not have the employer-employee data that would contain information about eligibility for the JobKeeper program, we select the 3.5 million recipients of this payment based on their propensity for losing employment, including for those who are currently self-employed. Because the wage subsidy was only given to businesses with severe financial difficulties, and its intention was to retain jobs that would otherwise be at risk of disappearing, we assume the likelihood of receiving the benefit can be approximated by the immediate risk of losing the job.

We use a standard probit model (see Appendix Table 9 for the model specification) to estimate an individual’s propensity of losing their job between early March and early April, which is a unique period, with increasing Covid-19 cases but with minimal policy intervention. Such an estimate reflects the propensity of job loss under the economic shock without the influence of the JobKeeper program, which is endogenous to the economic situation of the business. The conditional probabilities are then applied to the SIH data for further analysis. To further refine the estimation,^7^ we use a common alignment algorithm to ensure the simulation will have the number of recipients that matches the industry-specific statistics published by the Australian Treasury (Li & O'Donoghue, 2014).

### Limitations and Assumptions

The method assumes that observed changes in the intensive and extensive margins of the labour market can be effectively described through the semi-parametric process. Controlling for the observed characteristics, the conditional income distribution remains largely stable other than the wage level adjustment reflected by the payroll information. This mimics the short-term inelasticity of the conditional wage rate due to contracts and labour law. While this assumption may be suitable for the short term, the medium- to long-term impact could be challenging to examine under the framework due to the complex behavioural responses. Therefore, we focus on the short-term impact of the pandemic in this paper and use the upper- and lower-bound estimates to provide general guidance on the expected direction of change.

On the policy modelling front, it should be noted that the JobKeeper scheme simulation requires approximations due to data limitations. First, certain eligibility criteria variables, such as the tenure and turnover information for the scheme, are not collected in either survey. This means eligibility is approximated by the observed characteristics, and therefore may contribute to a potential bias in certain subpopulation groups with more complex working trajectories. Therefore, our findings focus on the expected changes in the income distribution in the overall population rather than a specific population subgroup.

Second, the JobKeeper approximation assumes that the scheme covers those who are at risk between early March and early April. Changes in the macroeconomic environment and business behaviours could complicate the simulation. Furthermore, some at-risk workers may not be covered as the policy intended due to the eligibility criteria. Although the simulation is partially corrected through the alignment process, the interpretation of our results should focus on the short-term impact, where the changes in the macroeconomic and behavioural factors are limited.

Finally, as some temporary payments, including JobKeeper, were not necessarily paid in the same month as individuals became eligible due to administrative delays, multiple temporary welfare payments are annualised and combined in this analysis. This means the actual income volatility may be higher than indicated in the simulation, although the general pattern remains.

## Results

### Validation and Labour Market Trends

Before discussing the results from the estimated income distribution, we assess whether the proposed method can construct a satisfactory socio-economic distribution. For an evolving income distribution affected by a sudden shock to the labour market, it is expected that the estimated distribution would be sensitive to changes in labour market conditions, whereas the general demographic distribution should remain mostly constant. An excessive deviation from the baseline demographic profile would mean that the reweighting process had struggled to adjust the weights using observations available in the original survey data.

Appendix Table 10 reports the main demographic characteristics of the original SIH dataset, the up-to-date LLFS dataset and the constructed dataset based on the semi-parametric reweighting. The results from the nowcast model generally fall between the two datasets, suggesting a stable demographic profile. We also compare the changes in the simulated unemployment rate versus the official unemployment rate published by the ABS between February 2020 and June 2020, as outlined in Appendix Table 11. The model results, especially, the longitudinal change between March and April, indicate that the method can capture the changes in the employment distribution. In the baseline month (February), the difference between the simulated and the official unemployment rate is less than 0.1 percentage point. As each month’s distribution is estimated separately, variations also come from statistical uncertainties such as sampling errors and the use of the random numbers in the simulation. Throughout the paper, we report all estimates between February and June (with April to June as the Covid-affected period) to assess the robustness of the results.

As well as the unemployment rate results reported in Table 11, Appendix Tables 12 and 13 report additional key trends: changes in working hours and in the top income sources at the household level. The nowcast model clearly demonstrates a downward trend in the average number of working hours. As these working hours were reported as usual working hours, the actual working hours are likely to have been lower than these, especially in the initial months of the economic shocks. The model suggests a drop of between 6 and 13% in working hours, which is comparable to the drop of 6% to 10% reported in the seasonally adjusted ABS figures.

In terms of income, wage and business income, on average, only experience minor fluctuations when wage subsidies are included in the reported income. Investment income dropped, as expected, due to market volatility. Income from government payments experienced the largest change over the period. It increased by more than a quarter on average due to the increased unemployment benefit as well as other welfare payments. As welfare payments are heavily means-tested in Australia, the gains in welfare payments are mostly absorbed by middle- and low-income households, as discussed in the later sections of this paper.

### Changes in the Labour Market and Income Distribution

Table 2 reports the estimated average monthly household gross market income by pre-Covid quintiles of equivalised household disposable income in dollar amounts, and shows the percentage change compared with the pre-Covid period, taking into account the observed changes in labour force participation. The market income change shows the extent of the Covid-19 shock as well as the impact of the government wage subsidy program (JobKeeper) on pre-tax earnings. As the government wage subsidy was distributed through the employer as part of the wage, or directly to the self-employed, the subsidy is included in market income. In the pre-Covid period, the estimates show that gross household incomes were mostly stable. In May 2020, gross market income increased for most quintiles, with an increase of over 20% for Q1. Q5 was the only exception, where we observe an initial decline of gross income in April but a gradual recovery since then.

**Table 2:** Estimated household monthly market income (labour, business, investment) by pre-Covid equivalised household disposable income quintiles

		Monthly Gross Income (A$)					% Change Compared with February				
		Q1	Q2	Q3	Q4	Q5	Q1	Q2	Q3	Q4	Q5
Before Covid-19											
February		897	3508	7563	11,749	22,635					
March		889	3474	7548	11,738	22,660	− 0.9%	− 1.0%	− 0.2%	− 0.1%	0.1%
Nowcast Model											
April		1102	3737	7862	12,005	22,428	22.9%	6.5%	4.0%	2.2%	− 0.9%
May		1113	3767	7906	12,072	22,603	24.1%	7.4%	4.5%	2.7%	− 0.1%
June		1064	3664	7868	12,102	22,744	18.7%	4.5%	4.0%	3.0%	0.5%
Without Policy Response (lower estimates)											
April		704	2759	6081	9592	18,949	− 21.5%	− 21.4%	− 19.6%	− 18.4%	− 16.3%
May		743	2884	6117	9683	18,653	− 17.2%	− 17.8%	− 19.1%	− 17.6%	− 17.6%
June		695	2659	5927	9573	18,998	− 22.5%	− 24.2%	− 21.6%	− 18.5%	− 16.1%
Without Policy Response (upper estimates)											
April		888	3457	7561	11,754	22,277	− 0.9%	− 1.5%	0.0%	0.0%	− 1.6%
May		903	3479	7609	11,804	22,455	0.7%	− 0.8%	0.6%	0.5%	− 0.8%
June		869	3379	7570	11,806	22,591	− 3.2%	− 3.7%	0.1%	0.5%	− 0.2%

**Table 3:** Estimated contributions of Covid-19 income shock effect and policy response effect to changes in monthly household market income by pre-Covid equivalised disposable income quintiles

		Q1		Q2		Q3		Q4		Q5	
		Low	High	Low	High	Low	High	Low	High	Low	High
Income Shock Effect (A$)											
April		− 193	− 8	− 749	− 51	− 1483	− 2	− 2157	5	− 3686	− 358
May		− 154	7	− 624	− 29	− 1446	45	− 2067	55	− 3982	− 180
June		− 202	− 28	− 849	− 129	− 1636	7	− 2176	57	− 3637	− 43
Policy Response Effect (A$)											
April		214	398	281	979	301	1782	250	2412	152	3480
May		210	370	288	883	297	1789	268	2389	148	3951
June		196	369	285	1005	298	1940	296	2529	152	3746
Income Shock Effect (Percentage of pre-Covid income)											
April		− 21.5%	− 0.9%	− 21.4%	− 1.5%	− 19.6%	0.0%	− 18.4%	0.0%	− 16.3%	− 1.6%
May		− 17.2%	0.7%	− 17.8%	− 0.8%	− 19.1%	0.6%	− 17.6%	0.5%	− 17.6%	− 0.8%
June		− 22.5%	− 3.2%	− 24.2%	− 3.7%	− 21.6%	0.1%	− 18.5%	0.5%	− 16.1%	− 0.2%
Policy Response Effect (Percentage of pre-Covid income)											
April		23.8%	44.3%	8.0%	27.9%	4.0%	23.6%	2.1%	20.5%	0.7%	15.4%
May		23.4%	41.3%	8.2%	25.2%	3.9%	23.6%	2.3%	20.3%	0.7%	17.5%
June		21.8%	41.1%	8.1%	28.7%	3.9%	25.7%	2.5%	21.5%	0.7%	16.6%

**Table 4:** Estimated monthly equivalised household disposable income by pre-Covid equivalised disposable income quintiles

		Monthly Disposable Income (A$)					% Change Compared with February				
		Q1	Q2	Q3	Q4	Q5	Q1	Q2	Q3	Q4	Q5
Before Covid-19											
February		1873	2826	4006	5541	9997					
March		1864	2812	4007	5557	9922	− 0.5%	− 0.5%	0.0%	0.3%	− 0.7%
Nowcast Model											
April		2178	3059	4174	5648	9954	16.3%	8.2%	4.2%	1.9%	− 0.4%
May		2178	3052	4140	5604	9872	16.3%	8.0%	3.3%	1.1%	− 1.2%
June		2179	3054	4185	5685	10,033	16.4%	8.1%	4.5%	2.6%	0.4%
Without Policy Response (lower estimates)											
April		1805	2560	3397	4643	8516	− 3.6%	− 9.4%	− 15.2%	− 16.2%	− 14.8%
May		1807	2588	3377	4642	8291	− 3.5%	− 8.4%	− 15.7%	− 16.2%	− 17.1%
June		1812	2548	3361	4665	8524	− 3.3%	− 9.8%	− 16.1%	− 15.8%	− 14.7%
Without Policy Response (upper estimates)											
April		1882	2846	4049	5607	9935	0.5%	0.7%	1.1%	1.2%	− 0.6%
May		1880	2839	4022	5563	9858	0.4%	0.4%	0.4%	0.4%	− 1.4%
June		1889	2840	4061	5629	10,021	0.8%	0.5%	1.4%	1.6%	0.2%

**Table 5:** Estimated contributions of Covid-19 income shock effect and policy response effect to changes in monthly equivalised household disposable income by pre-Covid equivalised disposable income quintiles

		Q1		Q2		Q3		Q4		Q5	
		Low	High	Low	High	Low	High	Low	High	Low	High
Income Shock Effect (A$)											
April		− 68	9	− 266	20	− 609	43	− 898	66	− 1480	− 62
May		− 65	7	− 238	13	− 629	16	− 900	22	− 1705	− 138
June		− 61	16	− 278	14	− 645	55	− 876	87	− 1472	24
Policy Response Effect (A$)											
April		297	373	213	499	124	777	40	1005	19	1438
May		298	371	213	464	118	763	41	962	14	1581
June		291	368	214	507	124	824	56	1020	12	1509
Income Shock Effect (percentage of pre-Covid income)											
April		− 3.6%	0.5%	− 9.4%	0.7%	− 15.2%	1.1%	− 16.2%	1.2%	− 14.8%	− 0.6%
May		− 3.5%	0.4%	− 8.4%	0.4%	− 15.7%	0.4%	− 16.2%	0.4%	− 17.1%	− 1.4%
June		− 3.3%	0.8%	− 9.8%	0.5%	− 16.1%	1.4%	− 15.8%	1.6%	− 14.7%	0.2%
Policy Response Effect (percentage of pre-Covid income)											
April		15.8%	41.6%	7.5%	17.6%	3.1%	19.4%	0.7%	18.1%	0.2%	14.4%
May		15.9%	41.3%	7.5%	16.4%	2.9%	19.1%	0.7%	17.4%	0.1%	15.8%
June		15.5%	41.0%	7.6%	17.9%	3.1%	20.6%	1.0%	18.4%	0.1%	15.1%

**Table 6:** Changes in income inequality in Australia, 2020

		Feb	Mar	Apr	May	Jun
Gini of Market Income (ages 15–64)		0.539	0.543	0.536	0.541	0.557
Changes relative to February		–	0.005	− 0.002	0.002	0.018
Income Shock Effect (low impact)		–	0.005	0.016	0.020	0.036
Income Shock Effect (high impact)		–	0.005	0.107	0.109	0.129
Policy Effect (low impact)		–	0.000	− 0.018	− 0.018	− 0.018
Policy Effect (high impact)		–	0.000	− 0.109	− 0.107	− 0.112
Gini of Disposable Income (population)		0.329	0.329	0.308	0.309	0.313
Changes vs February		–	− 0.001	− 0.021	− 0.021	− 0.017
Income Shock Effect (low impact)		–	− 0.001	− 0.001	− 0.001	0.003
Income Shock Effect (high impact)		–	− 0.001	0.036	0.031	0.045
Policy Effect (low impact)		–	0.000	− 0.020	− 0.019	− 0.020
Policy Effect (high impact)		–	0.000	− 0.057	− 0.052	− 0.061

The increase in gross market incomes between April and June in the bottom quintiles can largely be attributed to the wage subsidy scheme, as shown by the estimates without policy responses, which is unsurprising given that the subsidy can sometimes exceed the usual wage for casual workers. In the lower bound estimates, where we assume that all jobs supported by the JobKeeper Payment and the welfare support schemes are gone, a significant decline in gross earning is seen throughout the distribution. In the upper bound estimates, where no JobKeeper-supported jobs are lost, the changes in gross income are generally less than a few percentage points. The actual impact of the wage subsidy on labour demand is likely somewhere between those two extreme estimates. The difference between the model estimates and the estimates without the policy change shows the substantial impact of the policy change, especially for households in Q1 and Q2. The variation between estimates for the post-Covid period tends to be small, suggesting the initial policy response and economic shock are the dominant factors for the change in income.

In line with the decomposition framework, as set out in the methodology section, we estimate the contributions from the income shock effect and the government policy response to the overall changes in income. Table 3 reports the estimates and the ranges derived from the counterfactuals. The term “Low” is used in the table to indicate the lower bound estimates and the term “High” for the upper bound estimates. As shown, the income shock effect, which corresponds to term A in Eq. (1), contributes to a maximum of a A$100 decline in gross income for the lower-income households to up to a few thousand dollars for the high-income households. The policy response effect, which corresponds to term B in Eq. (1), generally negates the decline induced by the income shock effect and contributes to an increase of a few hundred to a few thousand dollars a month to household market income. Overall, the income shock due to Covid-19 contributes a maximum of 16–23% of the decline in gross income depending on the quintile, and the policy response contributes up to 44% of the pre-Covid gross income for households in Q1, and up to 29% for households in other quintiles. The net effect is reported in Table 2, with the impact being highly heterogeneous across quintiles.

Table 4 reports the changes in equivalised household disposable income after childcare costs by pre-Covid quintiles in absolute amounts, and showing relative changes. We deduct the childcare costs from disposable income as the consumption of childcare services changed significantly due to Covid-19 and the government policy response. In general, we observe a similar pattern to the one shown in Table 3. The largest changes in equivalised disposable income levels are found in Q1 and Q2, with increases of around 16% and 8%, respectively. It should be noted that while the analysis shows an average improvement in disposable income in the lower quintiles, this does not suggest all families are better off. The heterogeneity of shocks means that some individuals may be better off than the group average, while others could be significantly worse off, especially those who lost their jobs.

The impact of the Covid-19 outbreak would have been greater if there had been no policy response. Disposable income could have contracted by 3–4% for Q1 and by up to 10% for Q2 in the worst-case scenario. Due to the inability of the higher income groups to access means-tested welfare payments, Q3–Q5 would have suffered more in terms of percentage changes due to job losses, although they would still have been better off in absolute terms. Across all results, the variations over time between April and June tend to be small. As each month is estimated independently, the stability of the numeric results is a welcome sign, suggesting the robustness of the results derived from the semi-parametric approach.

Table 5 provides the estimates of the contributions from Covid-19 and the policy response to changes in disposable income. Compared with the estimates of gross income shown in Table 4, the absolute dollar amount change is generally lower, suggesting the automatic stabilising effect of the existing tax and benefit system. The equivalised disposable income level for Q1 households would only have experienced minor changes with or without any direct policy response, as the existing welfare payments were an important source of income which were unaffected by the Covid-19 outbreak. Households in Q2 to Q5 would have experienced a greater reduction of income under a no policy response scenario. The income shock effect would account for up to 20% of the reduction in disposable income in the worst-case scenario. The policy response effect is a net positive for all quintiles, responsible for the increase in disposable incomes, and with Q1 households benefiting the most. The change in disposable income is dominated by the increased level of welfare payments for Q1 and the wage subsidy for Q2–Q5. Free childcare has only a limited impact, as it only affects the out-of-pocket portion of a service that is already heavily subsidised.

### Changes in Inequality

This section examines how the changes observed across the distribution are reflected in the changes in market income inequality (for all persons aged between 15 and 64) and disposable income inequality. The nowcast estimates in Table 6 suggest that gross income inequality was relatively stable over time except for June, where there was an almost 0.02 point increase compared with February. However, when decomposing the change into the income shock effect and the policy response effect, it becomes clear that the Covid-19 income shock would have caused increases in gross income inequality ranging between 0.016 and 0.13 Gini points in April–June relative to February. The impact, however, is almost entirely negated by the policy effect due to the introduction of the wage subsidy. This finding is robust across both the extreme estimates, which provide the bounds of the estimates for the policy and the income effects.

Compared with the relatively stable gross market income inequality due to wage subsidies, disposable income inequality shows an apparent reduction. Compared with February 2020, when the Gini was around 0.33, the Gini was hovering around 0.31 in April, May and June. A drop of 0.02 Gini points in a few months is substantial given that the annual change in disposable income inequality measured by the Gini coefficient in Australia is usually smaller than 0.01 (Li et al., 2021). For example, to provide a comparison, the change in the Gini coefficient between 2007 and 2009 was less than 0.012 over the two years. As expected, the policy contributed to the substantial reduction in income inequality, dominating the rise in inequality induced by the income shock, as evaluated by our low and high impact estimates. This downward change in income inequality is not unique to Australia. A similar policy impact during the Covid-19 crisis was found by O’Donoghue et al. (2020) and Beirne et al. (2020) in Ireland and Brewer and Tasseva (2021) in the UK. They found that wage subsidy schemes cushioned family income across the distribution. Coupled with the rules embedded in the tax-benefit systems, these schemes provided much-needed income protection.

## Conclusions

Similar to other countries, Covid-19 has had a significant impact on social and economic activities in Australia. Using the combined data from the monthly LLFS, administrative payroll information and the SIH, this paper proposes a method to reconstruct the income distribution semi-parametrically from incomplete data, and uses this method to estimate the impact of the Covid-19 outbreak and the policy response on the income distribution.

While Covid-19 increased unemployment, the wage subsidy initiative from the government was effective in increasing market incomes for households in the lowest and the second-lowest income quintile, with a stabilising effect for the families situated in the mid to high part of the income distribution. Inequality in gross market income was generally stable despite the significant shocks introduced by Covid-19. The relatively positive outcome of the market income changes is primarily due to the flat rate wage subsidy for part-time and long-term casual workers, who could receive an income exceeding their usual wage.

In terms of disposable income, we observe an increase in the living standards for most households except those in the wealthiest income quintile between February and June 2020, although the increase is most evident in the bottom quintile, showing a similar pattern to the change in gross income. The increase in the bottom quintile can be mostly attributed to the policy response, which also negates the income shock effects for other quintiles. Due to the significant rise in disposable income in the bottom quintile, Australia experienced a rapid drop in income inequality, reducing the Gini coefficient for equivalised disposable income from 0.33 to 0.31.

While our analyses do not imply the measures have sheltered the shock for every household in need, the overall change in the income distribution does suggest the measures are generally progressive, protecting those who are, on average, more vulnerable. Our results also suggest the change or the lack of change in the income distribution in the initial aftermath of the Covid-19 outbreak was heavily dependent on the temporary measures. This raises the question of the impact on the income distribution once these policies are replaced or withdrawn. Future research may consider comparing the estimates when new household data become available to examine the longer-term impact of the shocks. Additionally, it could be interesting to further investigate the persistence and the heterogeneities of the income shocks.

Our findings also shows the feasibility of the methodological framework with partially updated data in the case of Australia. This method can be applied to countries where data availability is more restricted, especially those with limited comprehensive surveys but with more frequent short surveys. If the post-Covid data on behaviours of individuals and firms become available, the framework may also be used to estimate the impact of the withdrawal of the policies. Adopting this method in a cross-country context could also help to analyse whether the same tax-benefit policies may lead to similar results in different contexts, which could offer potential insights for both Covid-19 and general economic policy research.

## Appendix 1

See Tables 7, 8, 9, 10, 11, 12, 13.

**Table 7:** Probit model results used in reweighting between SIH and LLFS

Independent Variables	Feb		Mar		Apr		May		Jun	
	Coeff	Std Err	Coeff	Std Err	Coeff	Std Err	Coeff	Std Err	Coeff	Std Err
Age (capped at 84)	0.045	(0.051)	0.003	(0.057)	0.023	(0.062)	0.009	(0.056)	0.011	(0.062)
Age squared	− 0.000	(0.001)	0.000	(0.001)	− 0.000	(0.001)	0.000	(0.001)	− 0.000	(0.001)
Age 85 or above^a,b^	0.145^*^	(0.081)	0.096	(0.081)	− 0.032	(0.081)	− 0.069	(0.082)	0.029	(0.082)
Married	− 0.018	(0.022)	− 0.008	(0.022)	− 0.020	(0.022)	− 0.035	(0.022)	− 0.027	(0.023)
Male	0.444	(0.372)	0.672	(0.421)	0.674	(0.445)	0.656^*^	(0.397)	0.684	(0.432)
Male & married	− 0.026	(0.027)	− 0.018	(0.027)	0.008	(0.027)	0.000	(0.027)	− 0.002	(0.028)
Employment status^b,c^ (Ref: Full-time)										
- Part-time	− 0.385	(0.291)	− 0.649^**^	(0.293)	− 0.346	(0.303)	− 0.514^*^	(0.304)	− 0.419	(0.311)
- Unemployed	1.518	(1.140)	0.750	(1.303)	0.412	(1.390)	0.692	(1.275)	0.736	(1.360)
- Not in the labour force	0.645	(1.031)	0.240	(1.197)	0.906	(1.276)	0.705	(1.158)	1.021	(1.257)
Employment type^b,c^ (Ref: Not working & Family worker)										
Employee	0.403	(0.891)	0.401	(1.066)	− 0.197	(1.147)	0.276	(1.004)	0.472	(1.108)
Owner of incorporated business	1.106	(0.950)	0.989	(1.121)	0.626	(1.205)	1.024	(1.066)	1.145	(1.179)
Owner of unincorporated business	1.429	(0.924)	1.250	(1.093)	0.895	(1.173)	1.373	(1.031)	1.515	(1.139)
Education level^b,c^ (Ref: Postgraduate)										
Bachelor	− 0.045	(0.186)	0.044	(0.189)	0.063	(0.191)	0.039	(0.190)	− 0.009	(0.191)
TAFE	− 0.166	(0.172)	− 0.093	(0.176)	− 0.081	(0.178)	− 0.134	(0.178)	− 0.210	(0.179)
Year 12	− 0.142	(0.179)	− 0.045	(0.183)	0.002	(0.185)	− 0.009	(0.184)	− 0.045	(0.185)
Year 11 and below	0.097	(0.166)	0.172	(0.170)	0.114	(0.172)	0.070	(0.171)	0.017	(0.172)
Industry^b,c^ (Ref: Not working & Other services)										
Agri., Forestry & Fishing	− 0.234	(0.473)	− 0.187	(0.483)	− 0.163	(0.495)	− 0.114	(0.483)	− 0.391	(0.514)
Mining	0.513	(0.799)	0.924	(0.827)	1.333	(0.843)	0.582	(0.853)	0.868	(0.878)
Manufacturing	0.326	(0.433)	0.296	(0.435)	0.202	(0.447)	− 0.006	(0.448)	− 0.061	(0.461)
Elect., Gas, Water & Waste Serv	1.212	(0.884)	0.276	(0.875)	0.281	(0.907)	0.233	(0.882)	0.278	(0.907)
Construction	0.604	(0.425)	0.573	(0.430)	0.704	(0.438)	0.548	(0.435)	0.398	(0.451)
Wholesale Trade	1.209^**^	(0.557)	0.967^*^	(0.567)	1.225^**^	(0.570)	0.960^*^	(0.586)	0.930	(0.609)
Retail Trade	0.284	(0.406)	0.189	(0.409)	0.237	(0.420)	0.024	(0.420)	0.026	(0.432)
Accommodation and Food Serv	0.520	(0.402)	0.274	(0.406)	0.480	(0.421)	0.194	(0.422)	0.218	(0.435)
Transport, Postal & Warehousing	0.547	(0.528)	0.264	(0.545)	0.090	(0.556)	− 0.260	(0.554)	− 0.127	(0.569)
Infor. Media and Telecom	− 0.378	(0.596)	− 0.662	(0.592)	− 0.688	(0.636)	− 1.317^**^	(0.641)	− 1.625^***^	(0.658)
Financial and Insurance Services	− 0.286	(0.600)	− 0.212	(0.609)	− 0.029	(0.619)	− 0.025	(0.602)	− 0.130	(0.622)
Rental, Hiring & Real Estate Serv	0.097	(0.611)	− 0.116	(0.613)	0.062	(0.615)	− 0.221	(0.630)	0.033	(0.641)
Prof., Scientific & Tech. Serv	0.193	(0.445)	0.111	(0.456)	0.032	(0.470)	− 0.127	(0.466)	− 0.082	(0.480)
Administrative & Support Serv	0.566	(0.524)	0.394	(0.532)	0.064	(0.549)	− 0.052	(0.540)	− 0.306	(0.563)
Public Administration and Safety	0.559	(0.469)	0.576	(0.479)	0.798^*^	(0.484)	0.542	(0.487)	0.510	(0.501)
Education and Training	0.406	(0.444)	0.277	(0.448)	0.347	(0.460)	− 0.022	(0.464)	0.237	(0.478)
Health Care & Social Assistance	0.139	(0.410)	0.163	(0.414)	0.350	(0.425)	0.279	(0.426)	0.156	(0.438)
Arts and Recreation Services	− 0.175	(0.502)	− 0.220	(0.510)	-0.638	(0.540)	− 1.735^***^	(0.567)	− 1.566^***^	(0.576)
Occupation^b,c^ (Ref: Not working & Labourers)										
Managers	− 0.395	(0.311)	− 0.379	(0.320)	− 0.379	(0.325)	− 0.488	(0.320)	− 0.406	(0.333)
Professionals	− 0.338	(0.285)	− 0.344	(0.293)	− 0.230	(0.299)	− 0.382	(0.301)	− 0.509^*^	(0.311)
Technicians and Trades	− 0.577^**^	(0.281)	− 0.631^**^	(0.286)	− 0.465	(0.294)	− 0.575^**^	(0.292)	− 0.523^*^	(0.301)
Community & Personal Serv. Workers	− 0.010	(0.260)	− 0.183	(0.266)	− 0.477^*^	(0.277)	− 0.901^***^	(0.280)	− 0.665^**^	(0.285)
Clerical and Administrative Workers	− 0.373	(0.282)	− 0.352	(0.288)	− 0.290	(0.293)	− 0.183	(0.292)	− 0.312	(0.304)
Sales Workers	− 0.091	(0.254)	− 0.105	(0.260)	− 0.093	(0.263)	− 0.201	(0.265)	− 0.152	(0.273)
Machinery Operators and Drivers	− 0.828^**^	(0.371)	− 0.733^*^	(0.381)	− 0.510	(0.385)	− 0.321	(0.385)	− 0.387	(0.400)
Usual hours of working^b,c^ (Ref: Under 10 h/week)										
10–19 h/week	0.456^**^	(0.229)	0.725^***^	(0.235)	0.654^***^	(0.240)	0.641^***^	(0.241)	0.618^***^	(0.249)
20–29 h/week	0.298	(0.243)	0.313	(0.248)	0.323	(0.251)	0.168	(0.256)	0.208	(0.262)
30– 9 h/week	0.689^**^	(0.300)	0.374	(0.303)	0.494	(0.312)	0.251	(0.313)	0.419	(0.323)
40–49 h/week	0.084	(0.374)	− 0.215	(0.379)	0.022	(0.389)	− 0.308	(0.390)	− 0.275	(0.402)
50–59 h/week	− 0.075	(0.440)	− 0.628	(0.452)	− 0.387	(0.466)	− 0.607	(0.464)	− 0.736	(0.479)
60 + hours/week	0.269	(0.487)	0.099	(0.496)	0.386	(0.507)	0.141	(0.506)	0.086	(0.525)
Having only one job^b,c^	0.362	(0.251)	0.173	(0.252)	0.817^***^	(0.269)	0.664^***^	(0.265)	0.677^***^	(0.273)
Unemployment duration^b,c^ (Ref: Not unemployed)										
1–13 weeks	− 0.453	(0.575)	− 0.192	(0.597)	0.063	(0.632)	− 0.890	(0.618)	− 0.610	(0.607)
14–26 weeks	− 0.993	(0.732)	− 0.410	(0.740)	0.031	(0.775)	− 0.679	(0.756)	0.034	(0.728)
27–52 weeks	− 1.959^***^	(0.755)	− 1.069	(0.786)	− 0.730	(0.835)	− 1.496^*^	(0.806)	− 0.208	(0.768)
Foreign born^b,c^	0.011	(0.067)	− 0.000	(0.067)	0.017	(0.067)	0.021	(0.067)	0.027	(0.067)
Interaction between age &										
Employment status	YES		YES		YES		YES		YES	
Employment type	YES		YES		YES		YES		YES	
Education level	YES		YES		YES		YES		YES	
Industry	YES		YES		YES		YES		YES	
Occupation	YES		YES		YES		YES		YES	
Usual hours of working	YES		YES		YES		YES		YES	
Having a job	YES		YES		YES		YES		YES	
Unemployment duration	YES		YES		YES		YES		YES	
Foreign born	YES		YES		YES		YES		YES	
Interaction between age^2^ &	YES		YES		YES		YES		YES	
Employment status	YES		YES		YES		YES		YES	
Employment type	YES		YES		YES		YES		YES	
Education level	YES		YES		YES		YES		YES	
Industry	YES		YES		YES		YES		YES	
Occupation	YES		YES		YES		YES		YES	
Usual hours of working	YES		YES		YES		YES		YES	
Having a job	YES		YES		YES		YES		YES	
Unemployment duration	YES		YES		YES		YES		YES	
Foreign born	YES		YES		YES		YES		YES	
Interaction between female &	YES		YES		YES		YES		YES	
Employment status	YES		YES		YES		YES		YES	
Employment type	YES		YES		YES		YES		YES	
Education level	YES		YES		YES		YES		YES	
Industry	YES		YES		YES		YES		YES	
Occupation	YES		YES		YES		YES		YES	
Usual hours of working	YES		YES		YES		YES		YES	
Having a job	YES		YES		YES		YES		YES	
Unemployment duration	YES		YES		YES		YES		YES	
Foreign born	YES		YES		YES		YES		YES	
Interaction between male &	YES		YES		YES		YES		YES	
Employment status	YES		YES		YES		YES		YES	
Employment type	YES		YES		YES		YES		YES	
Education level	YES		YES		YES		YES		YES	
Industry	YES		YES		YES		YES		YES	
Occupation	YES		YES		YES		YES		YES	
Usual hours of working	YES		YES		YES		YES		YES	
Having a job	YES		YES		YES		YES		YES	
Unemployment duration	YES		YES		YES		YES		YES	
Foreign born	YES		YES		YES		YES		YES	
Number of children aged 0–4	0.006	(0.014)	0.005	(0.014)	− 0.009	(0.015)	0.001	(0.015)	0.009	(0.015)
Number of children aged 5–14	0.055^***^	(0.009)	0.053^***^	(0.009)	0.043^***^	(0.009)	0.032^***^	(0.009)	0.036^***^	(0.009)
Number of families in the household (Ref: Three or more)										
One	− 0.053^**^	(0.022)	− 0.047^**^	(0.023)	− 0.018	(0.023)	0.010	(0.023)	0.012	(0.023)
Two	− 0.116^***^	(0.043)	− 0.144^***^	(0.043)	− 0.033	(0.043)	0.004	(0.043)	− 0.013	(0.044)
State/territory of residence (Ref: New South Wales)										
Victoria	0.007	(0.017)	0.009	(0.017)	0.013	(0.017)	0.010	(0.017)	0.015	(0.017)
Queensland	0.006	(0.018)	0.009	(0.019)	0.017	(0.019)	0.017	(0.019)	0.019	(0.019)
South Australia	− 0.015	(0.019)	− 0.012	(0.019)	− 0.006	(0.019)	− 0.012	(0.019)	− 0.011	(0.019)
Western Australia	0.009	(0.020)	0.009	(0.020)	0.021	(0.020)	0.014	(0.020)	0.015	(0.020)
Tasmania	− 0.016	(0.020)	− 0.013	(0.020)	− 0.009	(0.021)	− 0.012	(0.021)	− 0.004	(0.021)
Northern Territory	0.067^**^	(0.031)	0.064^**^	(0.031)	0.080^***^	(0.032)	0.072^**^	(0.031)	0.067^**^	(0.032)
Australian Capital Territory	− 0.015	(0.027)	− 0.011	(0.027)	− 0.009	(0.027)	− 0.011	(0.027)	− 0.010	(0.028)
Constant	− 0.686	(1.041)	− 0.307	(1.207)	− 0.833	(1.286)	− 0.559	(1.169)	− 0.913	(1.267)
Observations	75,498	73,656	72,425	73,246	68,496					

Standard errors in parentheses. Models are estimated using standard probit with the independent variables listed in the table. Estimation sample includes all LLFS and SIH observations living in private dwellings. The dependent variable is coded as 1 for LLFS observations and 0 for SIH observations

a Age 85 or above variable is included to match the age cap of the basic version of the SIH dataset

b Variables include interaction terms with gender

c Variables include interactions terms with age and age squared

^*^ *p* <  = 0.10, ^**^ *p* <  = 0.05, ^***^ *p* <  = 0.01

**Table 8:** Probit model specification used in estimating the propensity of being employed at least once since February 2020

Independent Variables	Mar		Apr		May		Jun	
	Coeff	Std Err	Coeff	Std Err	Coeff	Std Err	Coeff	Std Err
Male	0.147	(0.235)	0.182	(0.200)	0.268	(0.221)	0.035	(0.262)
Married	0.060	(0.067)	0.069	(0.054)	0.020	(0.058)	0.099	(0.067)
Age	0.057^***^	(0.006)	0.062^***^	(0.005)	0.071^***^	(0.005)	0.064^***^	(0.006)
Age squared	− 0.001^***^	(0.000)	− 0.001^***^	(0.000)	− 0.001^***^	(0.000)	− 0.001^***^	(0.000)
Education level (Ref: Postgraduate)								
Bachelor	− 0.003	(0.118)	0.127	(0.109)	0.327^***^	(0.117)	0.228^*^	(0.133)
TAFE	− 0.168	(0.114)	0.039	(0.105)	0.019	(0.114)	− 0.059	(0.128)
Year 12	− 0.133	(0.114)	0.145	(0.104)	0.171	(0.113)	− 0.049	(0.129)
Year 11 and below	− 0.480^***^	(0.109)	− 0.360^***^	(0.104)	− 0.172	(0.110)	− 0.244^**^	(0.123)
Foreign born	0.171^***^	(0.063)	0.154^***^	(0.053)	0.161^***^	(0.055)	0.202^***^	(0.066)
Number of children aged 0–4	− 0.153^**^	(0.063)	− 0.298^***^	(0.054)	− 0.301^***^	(0.057)	− 0.174^***^	(0.063)
Number of children aged 5–14	− 0.136^***^	(0.042)	− 0.100^***^	(0.033)	− 0.132^***^	(0.034)	− 0.123^***^	(0.042)
State/territory of residence (Ref: New South Wales)								
Victoria	0.155^*^	(0.083)	0.018	(0.067)	0.024	(0.071)	0.087	(0.082)
Queensland	0.109	(0.092)	0.018	(0.073)	0.016	(0.076)	− 0.066	(0.092)
South Australia	0.004	(0.106)	0.011	(0.081)	− 0.008	(0.087)	0.052	(0.101)
Western Australia	0.144	(0.099)	0.077	(0.079)	0.093	(0.085)	0.067	(0.106)
Tasmania	0.280^***^	(0.110)	0.016	(0.095)	0.133	(0.144)	0.042	(0.113)
Northern Territory	0.328^*^	(0.184)	0.252^*^	(0.146)	0.201	(0.164)	0.232	(0.200)
Australian Capital Territory	0.123	(0.173)	0.166	(0.146)	0.214	(0.149)	0.237	(0.172)
Male & married	0.208^**^	(0.107)	0.214^**^	(0.089)	0.268^***^	(0.094)	0.241^**^	(0.107)
Interaction between male &								
Age	0.013	(0.010)	0.011	(0.007)	− 0.000	(0.007)	0.011	(0.009)
Age squared	− 0.000	(0.000)	− 0.000	(0.000)	0.000	(0.000)	− 0.000	(0.000)
Education level (Ref: Postgraduate)								
Bachelor	− 0.056	(0.181)	− 0.265	(0.170)	− 0.327^*^	(0.190)	− 0.197	(0.222)
TAFE	− 0.077	(0.167)	− 0.292^*^	(0.157)	− 0.140	(0.179)	− 0.015	(0.208)
Year 12	− 0.335^*^	(0.177)	− 0.442^***^	(0.161)	− 0.371^**^	(0.182)	− 0.237	(0.216)
Year 11 and below	− 0.136	(0.167)	− 0.227	(0.157)	− 0.273	(0.177)	− 0.153	(0.206)
Foreign born	− 0.211^**^	(0.095)	− 0.225^***^	(0.080)	− 0.225^***^	(0.084)	− 0.134	(0.099)
Number of children aged 0–4	0.116	(0.120)	0.147	(0.111)	0.169	(0.117)	0.125	(0.131)
Number of children aged 5–14	0.167^***^	(0.066)	0.049	(0.054)	0.199^***^	(0.056)	0.190^***^	(0.067)
State/territory of residence (Ref: New South Wales)								
Victoria	− 0.208^*^	(0.120)	0.002	(0.101)	-0.075	(0.108)	− 0.099	(0.124)
Queensland	− 0.217	(0.134)	0.037	(0.110)	0.012	(0.113)	0.089	(0.136)
South Australia	− 0.470^***^	(0.166)	− 0.120	(0.122)	− 0.137	(0.130)	− 0.225	(0.155)
Western Australia	− 0.093	(0.144)	0.013	(0.122)	− 0.130	(0.129)	− 0.254	(0.162)
Tasmania	− 0.381^**^	(0.160)	− 0.104	(0.145)	− 0.055	(0.179)	− 0.021	(0.168)
Northern Territory	− 0.159	(0.249)	− 0.235	(0.234)	− 0.014	(0.238)	− 0.107	(0.293)
Australian Capital Territory	− 0.261	(0.256)	− 0.228	(0.227)	− 0.272	(0.219)	− 0.425	(0.263)
Constant	− 2.055^***^	(0.145)	− 1.620^***^	(0.127)	− 1.672^***^	(0.137)	− 1.698^***^	(0.158)
Observations	13,717	12,115	10,016	7509				

Standard errors in parentheses. Models are estimated using standard probit with the independent variables listed in the table. For March, April, May and June estimates, the dependent variable is coded as 1 if the person was in employment in any wave of the survey between February and the month of the estimation, and 0 otherwise. Only individuals who were out of the labour force in the month of the estimation are included. All variables are interacted with gender to capture the heterogeneous effect by gender. Detailed coefficient tables are available on request

^*^ *p* <  = 0.10, ** *p* <  = 0.05, *** *p* <  = 0.01

**Table 9:** Probit model specification used in estimating the propensity of remaining in employment conditional on being previously employed (March to April, under Covid-19 but limited policy intervention)

Independent Variables	Female		Male	
	Coeff	Std Err	Coeff	Std Err
Age	0.026^**^	(0.012)	0.017	(0.011)
Age squared	− 0.000^*^	(0.000)	− 0.000^**^	(0.000)
Age 85 or above^a^	0.000	(.)	− 1.238^**^	(0.606)
Married	0.042	(0.075)	0.190^**^	(0.086)
Part-time employed	− 0.298^***^	(0.095)	− 0.203^*^	(0.115)
Employment type (Ref: Family worker)				
Employee	− 0.133	(0.107)	− 0.004	(0.094)
Owner of incorporated business	− 0.496^***^	(0.079)	− 0.067	(0.080)
Owner of unincorporated business	− 1.189^***^	(0.239)	− 0.568	(0.400)
Education level (Ref: Postgraduate)				
Bachelor	0.056	(0.088)	0.059	(0.098)
TAFE	− 0.034	(0.095)	0.078	(0.104)
Year 12	− 0.089	(0.101)	0.084	(0.111)
Year 11 and below	0.032	(0.106)	0.126	(0.113)
Industry (Ref: Agri., Forestry & Fishing)				
Mining	0.190	(0.307)	− 0.210	(0.181)
Manufacturing	0.476^***^	(0.188)	0.154	(0.158)
Elect., Gas, Water & Waste Serv	− 0.021	(0.285)	0.074	(0.251)
Construction	0.309	(0.200)	− 0.027	(0.150)
Wholesale Trade	0.146	(0.207)	0.207	(0.195)
Retail Trade	0.453^***^	(0.167)	0.192	(0.166)
Accommodation and Food Serv	0.523^***^	(0.164)	0.090	(0.163)
Transport, Postal & Warehousing	0.168	(0.209)	0.046	(0.165)
Infor. Media and Telecom	0.245	(0.268)	0.547^*^	(0.300)
Financial and Insurance Services	0.353^*^	(0.201)	0.630^**^	(0.260)
Rental, Hiring & Real Estate Serv	0.333	(0.207)	0.147	(0.242)
Prof., Scientific & Tech. Serv	0.435^***^	(0.162)	− 0.154	(0.162)
Administrative & Support Serv	0.416^**^	(0.180)	0.029	(0.187)
Public Administration and Safety	0.386^**^	(0.185)	0.353^**^	(0.182)
Education and Training	0.622^***^	(0.170)	0.032	(0.179)
Health Care & Social Assistance	0.497^***^	(0.155)	0.283	(0.182)
Arts and Recreation Services	0.420^**^	(0.207)	0.235	(0.219)
Other services	0.571^***^	(0.187)	0.163	(0.190)
Occupation (Ref: Managers)				
Professionals	− 0.046	(0.109)	0.171	(0.107)
Technicians and Trades	− 0.037	(0.144)	0.017	(0.093)
Community & Personal Serv. Workers	− 0.038	(0.117)	− 0.026	(0.126)
Clerical and Administrative Workers	− 0.067	(0.106)	0.123	(0.127)
Sales Workers	− 0.076	(0.128)	0.069	(0.130)
Machinery Operators and Drivers	− 0.056	(0.216)	− 0.054	(0.107)
Labourers	− 0.060	(0.128)	0.007	(0.099)
Usual hours of working (Ref: under 10 h/week)				
10–19 h/week	0.493^***^	(0.087)	0.342^***^	(0.111)
20–29 h/week	0.748^***^	(0.091)	0.534^***^	(0.112)
30–39 h/week	0.676^***^	(0.105)	0.696^***^	(0.134)
40–49 h/week	0.586^***^	(0.134)	0.799^***^	(0.150)
50–59 h/week	0.520^***^	(0.184)	0.828^***^	(0.170)
60 + hours/week	0.605^***^	(0.220)	0.848^***^	(0.177)
Having more than one job	0.478^***^	(0.135)	0.256^*^	(0.147)
Foreign born	0.241^***^	(0.054)	0.088	(0.055)
Number of children aged 0–4	− 0.051	(0.053)	0.043	(0.062)
Number of children aged 5–14	0.037	(0.035)	0.005	(0.035)
Number of families in the household (Ref: Three or more)				
One	0.057	(0.096)	0.008	(0.090)
Two	− 0.009	(0.159)	0.009	(0.165)
State/territory of residence (Ref: New South Wales)				
Victoria	− 0.092	(0.067)	0.093	(0.067)
Queensland	0.003	(0.076)	0.150^**^	(0.073)
South Australia	0.070	(0.090)	0.292^***^	(0.093)
Western Australia	− 0.002	(0.084)	0.037	(0.077)
Tasmania	− 0.131	(0.094)	0.096	(0.095)
Northern Territory	− 0.041	(0.139)	0.117	(0.130)
Australian Capital Territory	0.185	(0.145)	0.279^*^	(0.152)
Constant	0.235	(0.323)	0.621^**^	(0.320)
Observations	11,942	13,095		

Standard errors in parentheses. Models are estimated using standard probit with the independent variables listed in the table. The dependent variable is coded as 1 if the person remains in employment in April 2020, and 0 if the person is no longer employed. Estimation sample includes observations who were employed in the previous month, estimated separately for male and female, and each wave of the LLFS. Only individuals who were working in March 2020 are included in the estimation. ^*^ *p* <  = 0.10, ^**^ *p* <  = 0.05, ^***^ *p* <  = 0.01

a Age 85 or above variable is included to match the age cap of the basic version of the SIH dataset

**Table 10:** Comparison between modelled and observed demographic profile among population aged 15 or above (February 2020)

Variable	SIH	LLFS	Modelled
Proportion of population between 15 and 24	15.7%	15.7%	16.0%
Proportion of population between 25 and 64	65.7%	65.3%	66.6%
Proportion of population 65 +	18.6%	19.0%	17.4%
Proportion of male	49.0%	49.2%	49.4%
Proportion of married (incl. de facto)	61.0%	59.5%	59.8%
Australian born	65.9%	67.0%	67.1%
Education bachelor or higher	27.8%	28.6%	29.0%
Number of children under 15 in the household	0.52	0.54	0.55

**Table 11:** Modelled unemployment rates compared with official figures, 2020

	Unemployment Rate		Youth Unemployment Rate	
	Modelled	Official	Modelled	Official
Feb	5.45%	5.52%	13.14%	13.18%
Mar	5.69%	5.57%	13.74%	12.64%
Apr	6.66%	6.43%	15.21%	14.21%
May	6.90%	6.92%	15.20%	15.19%
Jun	8.66%	7.25%	18.88%	15.64%

**Table 12:** Changes in average weekly working hours in 2020 (Age 15 +)

Month	Usual Hours of Working	Change Compared with February
Feb	22.5	–
Mar	21.9	− 2.4%
Apr	21.1	− 6.0%
May	21.1	− 6.2%
Jun	19.7	− 12.6%

**Table 13:** Changes in working hours and major income sources in 2020

		Feb	Mar	Apr	May	Jun
Nowcast Household Income Estimates (A$)						
Wage income		6269	6223	6345	6359	6030
Business Income		439	426	446	458	437
Investment Income		673	679	585	613	626
Government payments^a^		786	804	995	1002	1069
Changes compared with February						
Wage income		–	− 0.7%	1.2%	1.4%	− 3.8%
Business Income		–	− 2.9%	1.6%	4.3%	− 0.5%
Investment Income		–	0.9%	− 13.0%	− 9.0%	− 7.0%
Government payments^a^		–	2.3%	26.5%	27.5%	36.0%

^a^Government payments exclude childcare subsidies, as neither the services nor the fees are directly comparable before and after the Covid-19 restrictions

## Footnotes

1 For a complete timeline of the Australian government response, please refer to Campbell and Vines (2021).

2 As March can be considered a transitory month in terms of both policy measures and the propagation of the economic shocks, we generally do not interpret the results from March.

3 We use Gini coefficient in this paper due to its popularity and the ease of comparisons with related literature. However, other inequality indexes such as percentile ratios, Atkinson index and generalized entropy measures, can also be estimated under this framework.

4 The estimation of Eq. (1) can also be done using a different path of decomposition, where $I\left({p}_{1},{y}_{0}\right)$ can be used as a counterfactual instead of $I\left({p}_{0},{y}_{1}^{*}\right)$. This is sometimes referred as the path dependency issue. However, the measure $I\left({p}_{1},{y}_{0}\right)$ does not have a meaningful interpretation, as the Covid-19 policy response will exist only when Covid-19 occurs. We therefore adopt the sequential decomposition approach as described in the main text.

5 Due to an administrative delay, the first payment of JobKeeper was provided to eligible employers in the first week of May although the scheme covered eligible employees from 30 March 2020. For consistency,, we attribute the payment based on the matching employment period rather than the payment date.

6 Please refer to https://stinmod.canberra.edu.au for more information on STINMOD + .

7 It should be noted that the distribution from the unconstrained simulation already shows a good resemblance to the distribution from the official statistics. The Pearson correlation coefficient between the simulated and the actual number of recipients by 20 industry groups exceeds 0.8 prior to the incorporation of the industry-specific alignment. This result suggests that the approach of approximating the JobKeeper scheme with the at-risk group can be considered reasonable.
